# Evaluation of sample preparation methods for NMR-based metabolomics of Atlantic salmon (*Salmo salar*) ovarian fluid

**DOI:** 10.1007/s10695-026-01678-0

**Published:** 2026-04-15

**Authors:** Fabio Casu, Amanda L. Bayless, Brian C. Peterson, Heather J. Hamlin, Ashley S. P. Boggs, Tracey B. Schock

**Affiliations:** 1 Chemical Sciences Division, National Institute of Standards and Technology (NIST), Hollings Marine Laboratory, Charleston, SC 29412 USA; 2 USDA Agriculture Research Service, National Cold Water Marine Aquaculture Center, 25 Salmon Farm Road, Franklin, ME 04634 USA; 3 Aquaculture Research Institute, University of Maine, 17 Godfrey Dr., Orono, ME 04473 USA; 4 School of Marine Sciences, University of Maine, 360 Aubert Hall, Orono, ME 04469 USA

**Keywords:** Atlantic salmon, *Salmo salar*, Ovarian fluid, Method development, NMR metabolomics, Aquaculture

## Abstract

Average embryo survival in farmed Atlantic salmon (*Salmo salar*) has declined sharply from about 80% to below 50% in North America over the past two decades. The underlying causes remain unclear, and no effective solutions currently exist for this problem, posing a major challenge to the production of a leading aquaculture species in the USA. Traditional selective breeding programs typically assign “breeding values” to broodstock fish based on growth-related traits; however, incorporating reproductive success traits (e.g., embryo hatch rates, metabolic biomarkers) into breeding value calculations may improve the efficiency of broodstock selection and help identify individuals more likely to produce viable offspring. Ovarian fluid (OF), collected non-lethally during egg harvesting, offers a valuable biological matrix for metabolomics-based biomarker discovery for fish reproduction. This study tested seven sample preparation methods for nuclear magnetic resonance (NMR) metabolomics analysis of OF: (a) *filtration*; (b) *protein precipitation*; (c) *lyophilization*; (d) *dilution*; (e) *lyophilization-filtration*; (f) *dilution-filtration*; (g) *filtration-concentration*. The objectives were to confirm that OF contains measurable metabolites, and to establish a repeatable and practical OF sample preparation method for NMR metabolomic analysis. Among the methods tested, *filtration-concentration* produced the most consistent quality spectra with lower variability, higher signal-to-noise ratio, and ease of execution compared with alternative methods tested. In addition, this study demonstrates that blood contamination that may accidentally occur during ovarian fluid collection can significantly alter OF metabolite profiles, potentially affecting downstream analyses and data interpretation. These findings provide a foundation for standardized NMR metabolomics in salmon broodstock reproductive research.

**Supplementary information:**

The online version contains supplementary material available at 10.1007/s10695-026-01678-0.

## Introduction

Over the past 25 years, the hatch rate of farmed Atlantic salmon (*Salmo salar*) embryos in North America has seen a steady decline from approximately 80 to 90% in the early 2000 s to less than 50% with the underlying cause(s) of this dramatic decline still uncertain (Legacki et al. 2023; Thayer and Hamlin 2016). Viable solutions are lacking for an issue that is affecting the production of one of the top aquaculture finfish species in states such as Maine, a leading producer of this species in the USA (Legacki et al. 2023; Thayer and Hamlin 2016). Selective Atlantic salmon breeding programs, such as the North American Atlantic salmon breeding program developed at the USDA-ARS National Cold Water Marine Aquaculture Center in Franklin, ME, USA, have historically assigned a “breeding value” to individual broodstock fish with the goal of making informed and effective decisions on the selection of breeding pairs for the next generation. Breeding values are estimates of an individual’s genetic merit for specific traits and are calculated using statistical models that incorporate information from the individual and its relatives (full-siblings, half-siblings), as well as pedigree data. By selecting individuals with high breeding values for commercially important traits, these programs aim to increase the overall output of the production system and improve the overall performance of farmed salmon populations. Although breeding values at different salmon producers are based on proprietary formulas, growth parameters (e.g., carcass weight) are heavily weighted factors in these calculations. There are trade-offs between fish growth and fish reproduction and the relationship between a fish’s growth rate and its reproductive success can be quite complex; as a result, the best fish for growth are not necessarily the best for reproduction (Roff 1983; Folkvord et al. 2014). By incorporating measurements related to reproductive success (e.g., egg quality, fertilization success, embryo hatch rates, biomarkers) in breeding value calculations, breeding individuals with traits contributing to higher embryo survival can be identified and prioritized in selective breeding programs.

The use of cutting-edge molecular technologies can aid in the identification of biomarkers of superior reproductive fitness in Atlantic salmon through the analysis of tissues and/or biofluids collected from broodstock fish. One such sensitive analytical tool is metabolomics, one of the most recent “omics” technologies. Metabolomics is the study of the pool of low-molecular-weight (< 1500 Da) organic molecules or “metabolites” that constitute the so-called metabolome of an organism and its tissues and biofluids. Metabolites are the substrates, intermediates, and end products of gene expression and cellular metabolism given that they are the result of enzymatic and protein activities, and they are involved in a complex system of biochemical pathways that play crucial regulatory roles in biological systems (Fiehn 2002; Viant 2007; Gebregiworgis and Powers 2012). Compared with other “omics” technologies (genomics, transcriptomics, proteomics), metabolomics provides the closest representation of an organism’s molecular phenotype (Dunn et al. 2011; Fiehn 2002). The metabolome is highly sensitive to changes induced by different factors including genetic, environmental (including diet), and pathophysiological stimuli, and the responses to these stimuli are reflected in qualitative and/or quantitative changes in specific metabolites and therefore changes in an organism’s tissues/biofluids metabolome (Ramsden 2009; Nicholson et al. 1999). Metabolomics analysis relies on the use of high-resolution analytical platforms including mass spectrometry (MS) and nuclear magnetic resonance (NMR) spectroscopy. Specifically, NMR spectroscopy is a highly reproducible and inherently quantitative technique that allows the simultaneous detection and quantification of the metabolites present in a biological sample, including complex mixtures such as tissue extracts or biofluids (Markley et al. 2017; Nagana and Raftery 2019). Importantly, NMR spectroscopy is unbiased and can be applied to the analysis of compounds from a variety of chemical classes (e.g., amino acids, carbohydrates, nucleotides, organic acids, alkaloids). In addition, NMR spectroscopy is non-destructive in that the sample is not altered during analysis, and requires minimal sample preparation prior to instrumental analysis, without the need for chromatographic separation or chemical modification (Emwas et al. 2019; Nagana and Raftery 2021).

NMR-based metabolomics is increasingly being used in biomarker research with the goal of identifying biochemical markers that can be used for the early detection and diagnosis of human diseases, including cancer and neurological disorders (Gebregiworgis and Powers 2012; Emwas et al. 2016). In addition, NMR metabolomics offers a powerful approach to understanding the metabolic changes associated with various reproductive states and conditions in animals (Lombó et al. 2021; Gérard et al. 2015; Velho et al. 2017; Zhang et al. 2018). NMR-based metabolomics can be used as a discovery tool to analyze the metabolome and evaluate potential differences in metabolite profiles associated with an animal reproductive status (Gérard et al. 2015). In the case of fish, there are several limitations in the measurement of their metabolome, which are mainly based on time-consuming, costly, and invasive tissue sampling procedures for subsequent laboratory analyses. In fish research, several biofluids are commonly analyzed to understand physiological processes, and the impacts of environmental factors, disease states, and reproductive status; these include blood plasma (and serum), the collection of which is considered minimally invasive, as well as skin mucus and genital fluids (ovarian and seminal fluids) (Carriquiriborde 2020; Rosengrave et al. 2009). Among fish, female salmonids have a unique ability to retain ovulated oocytes (eggs) in their body cavity for several days post-ovulation without significant loss of viability. This is facilitated by the “coelomic” or “ovarian” fluid (OF), a semi-viscous biological fluid produced by filtration from the blood plasma and secreted by ovarian epithelia that surrounds and protects the maturing eggs and helps preserve their viability by providing the required nutrients (Gueho et al. 2024; Rosengrave et al. 2009). Fish OF has a complex biochemical composition that includes proteins, free amino acids, carbohydrates, and lipids; OF chemical composition varies between fish species, and is known to play key roles in egg maturation and fertilization success (Rosengrave et al. 2009; Nynca et al. 2022; Hirano et al. 1978; Lahnsteiner et al. 1995). Utilizing biofluids such as ovarian fluid that can be easily collected during the egg collection process can provide a non-lethal and relatively non-invasive sampling method and a valuable matrix to study the fish metabolome for fish reproduction. In its compositional complexity, biochemical compounds within the ovarian fluid metabolome can be evaluated for use as biomarkers of superior reproductive fitness when correlated to other biological parameters and metrics associated with reproductive success (e.g., embryo hatch rates).

Currently, there are no available analytical methods for the processing of fish ovarian fluid for NMR-based metabolomic analysis. The aim of this study was twofold: (1) to verify that ovarian fluid contains metabolites measurable by NMR spectroscopy, and (2) to establish a comprehensive, repeatable, and practical fish ovarian fluid processing method for NMR metabolomic analysis. To this aim, we tested seven sample preparation methods for NMR sample preparation: (a) *filtration*; (b) *protein precipitation*; (c) *lyophilization*; (d) *dilution*; (e) *lyophilization* + *filtration*; (f) *dilution* + *filtration*; and (g) *filtration* + *concentration*. *Filtration* uses 3-kDa molecular-weight-cutoff centrifugal filters that use size exclusion to remove macromolecules from the samples, including proteins, large lipids (e.g., lipoproteins), and nucleic acids that would interfere with the NMR analysis of small metabolites. The *protein precipitation* method is based on the addition of pre-chilled methanol at a 2:1 (v/v) ratio (methanol:ovarian fluid) to remove proteins and other macromolecules by precipitating them out of solution. The different methods were tested based on NMR spectral reproducibility, signal-to-noise (*S*/*N*) ratios, metabolite coverage, and practical ease. Due to the potential for blood contamination during ovarian fluid collection which could alter the metabolite profiles, we also evaluated the effects of two different levels of blood contamination (low and high) on the resulting NMR spectra for the different methods tested.

## Materials and methods

### Experimental fish husbandry and selection

Atlantic salmon (*Salmo salar*) for this study were reared at the USDA-ARS National Cold Water Marine Aquaculture Center (NCWMAC Franklin, ME, USA), in a recirculating aquaculture system. Atlantic salmon were held in 2 × 46 m^3^ tanks in natural light conditions, with a water flow rate of 4470 L/min and a makeup rate of 0.042 L/min, and were stocked at a maximum density of 40 kg/m^3^. Fish were held in high-salinity brackish well water (≈ 15 ppt) from 2 to 3 years of age. In August of 2022 at approximately 3 years of age, broodstock were transferred into fresh well water with an average salinity of 0.51 ± 0.09 ppt. Additional water quality parameters, monitored daily, were as follows: temperature (12.75 ± 0.49 °C), dissolved oxygen (DO, 70 to 120% saturation), pH (7.84 ± 0.12), total ammonia nitrogen (0.26 ± 0.11 mg/L), unionized ammonia (0.005 ± 0.002), nitrite (0.02 ± 0.01 mg/L), nitrate (0.61 ± 0.04 mg/L), CO_2_ (2.25 ± 0.48 ppm), and alkalinity (87 ± 8.14 ppm). Atlantic salmon were fed two to four times/day on a commercial diet with a pellet size of 6.0 to 12.0 mm. In early fall 2022, broodstock identified as likely to become sexually mature were separated by sex. Maturity likelihood was based on vent swelling, body conformation, and color. All identified females used in this study were defined as “ripe” (post-ovulation) based on visible abdominal swelling, extended vent, and eggs readily expressed through manual palpation, suggesting final oocyte maturation.

### Ovarian fluid collection and preparation

Ovarian fluid collection occurred from November to December 2022 at the NCWMAC (Franklin, ME, USA) according to the approved protocols at the USDA (IACUC Approval No 2022–06). Three-year-old Atlantic salmon were anesthetized in tricaine methane-sulfonate (MS-222, Syndel, Ferndale, WA, USA) at a concentration of 100 mg/L and buffered with sodium bicarbonate (Sigma-Aldrich, Inc, St. Louis, MO) for 15 min and removed from the holding tank for sampling. Each fish had a passive integrated transponder (PIT) tag number which was scanned before sampling for identification, and body weight was recorded before sample collection. Fish were wiped with towels to remove water from the vent and then strip-spawned by applying a gentle pressure and massaging in one motion from the dorsal area of the fish to the vent to facilitate the release of eggs and OF into a clean collection bucket. From each fish, two × 1.5-mL aliquots of OF (one for analysis and one for quality control (QC) material) were then transferred into 2.0-mL cryovials using a pipette and flash frozen in liquid nitrogen. The OF samples were placed in a liquid-nitrogen-vapor dry shipper for shipment to the National Institute of Standards and Technology (NIST) at the Hollings Marine Laboratory (HML, Charleston, SC, USA) where they were subsequently stored at − 80 °C until processing.

#### Ovarian fluid quality control (QC) material preparation

**Fig. 1:**
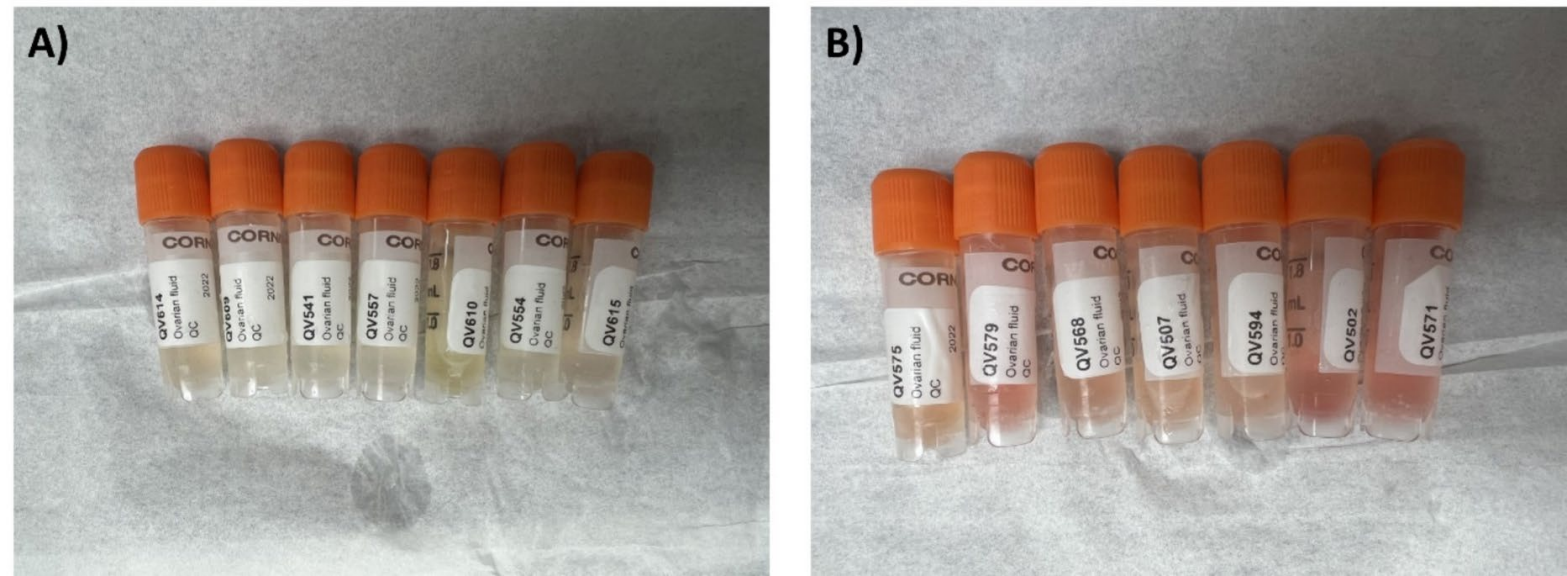
Atlantic salmon ovarian fluid quality control (QC) samples showing the variation in color from **A** yellow to **B** red for low to high suspected blood contamination levels, respectively

During sample collection, we observed variation in the color of the ovarian fluid, suspected to be blood contamination. To assess the effect of blood contamination levels on metabolite profiles, two separate pooled salmon ovarian fluid control materials were prepared using QC samples with different degrees of blood contamination based on visual inspection (low/yellow or high/red) (Fig. 1A and B).

**Fig. 2:**
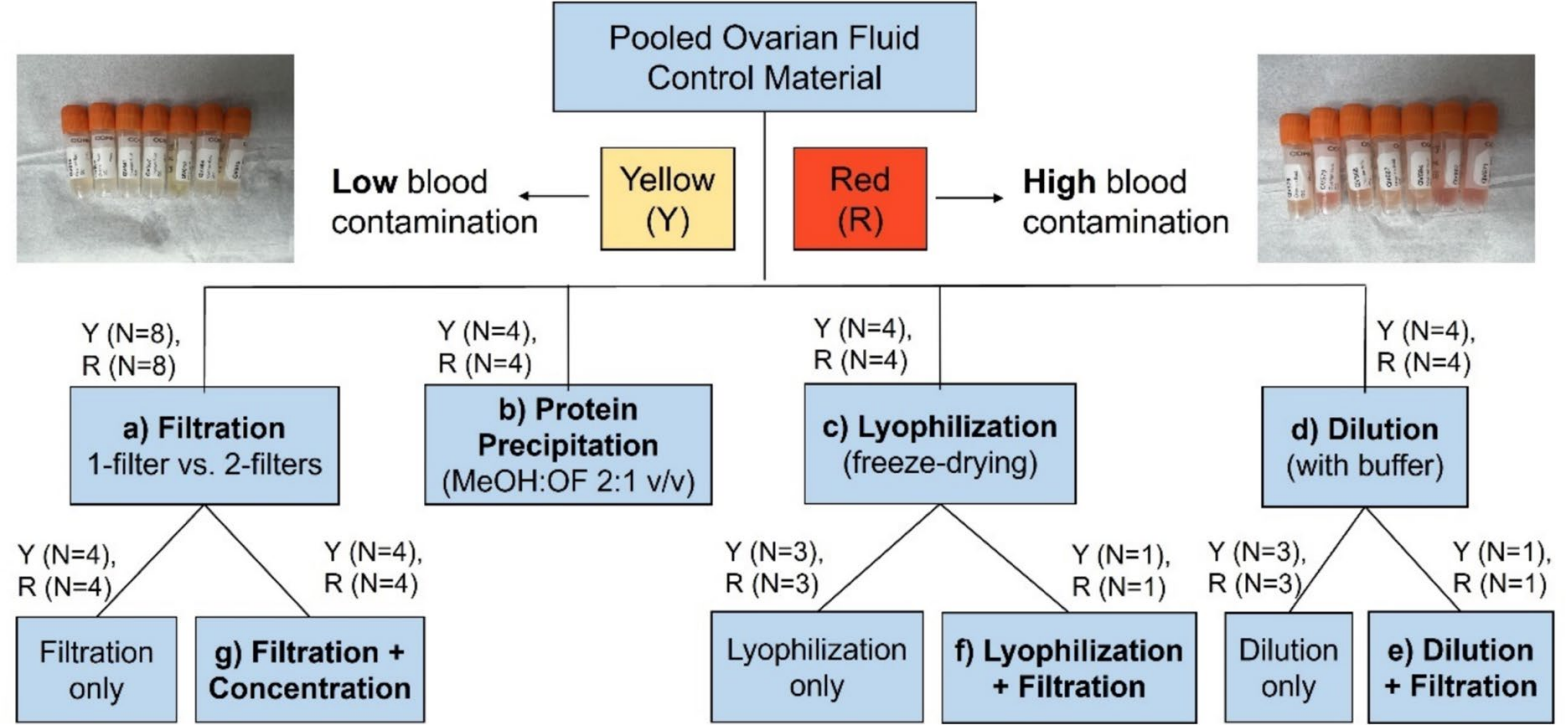
Overview of method development for Atlantic salmon ovarian fluid processing for NMR metabolomics analysis. Seven methods (**a**–**g**, in bold) were tested, in addition to three method variations in this study. Number of aliquots for the low (yellow, Y) and high (red, R) blood-contamination QC pools is indicated for each method

A total of 24 × 1.5-mL liquid-nitrogen-frozen ovarian fluid QC samples, 12 yellow and 12 red, were randomly selected from the overall population of QC samples. Upon thawing the samples on ice for ≈ 2.5 h, each separate pool was prepared by transferring 12 × 1.5-mL QC samples into a 50-mL Falcon tube and vortexing for 30 s. The two pooled materials were then aliquoted into 18 × 0.4-mL and 4 × 1-mL (22 total aliquots per pooled material), of which 20 aliquots (per pool) were used for this study (Fig. 2). Aliquots were stored at − 80 °C until analysis.

### Ovarian fluid processing methods for NMR metabolomics analysis

Seven different sample preparation methods were tested in this study (Fig. 2): (a) *filtration* (one filter and two filters/sample); (b) *protein precipitation*; (c) *lyophilization*; (d) *dilution*; (e) *dilution* + *filtration*; (f) *lyophilization* + *filtration*; (g) *filtration* + *concentration.* Samples were thawed on ice for ≈ 2.5 h prior to processing using each method. A total of 40 OF aliquots across all methods were analyzed by ^1^H NMR spectroscopy for this study. An overview of aliquots and sample volumes used for each preparation method is provided in Supplementary Table 1 (S1). Sample volumes for the filtration method were based on optimized biofluid volumes using 3-kDa MW cutoff centrifugal filters from previous studies conducted in our laboratory (Schock et al. 2013).

#### Filtration (one-filter and two-filter approaches)

Twelve Nanosep centrifugal filters (3-kDa MW cutoff) (Pall Life Sciences, Pall Corporation, Port Washington, NY, USA) were placed in a 1-L beaker with ≈ 800 mL MilliQ water and a magnetic stirring bar to wash overnight at room temperature under constant stirring to remove glycerol present in the filters (as indicated by the manufacturer’s product information), which would otherwise interfere with NMR analysis. The following morning pre-washed filters were centrifuged at 2000 × *g* at 25 °C for 5 min to remove residual water. Two approaches were compared to ensure an adequate volume of filtrate for NMR analysis: one filter per sample and two filters per sample. In the *one-filter approach*, ≈ 350 µL of ovarian fluid was loaded onto a centrifugal filter and centrifuged at 10,621 × *g* at 4 °C for ≈ 1.5 h to obtain ≈ 200 µL of filtrate. This filtrate was mixed with 400 µL of NMR phosphate buffer (100 mmol/L sodium phosphate in D_2_O (pH = 7.3, measured) containing 1.0 mmol/L sodium 3-trimethylsilyl 2,2,3,3-d_4_ propionate (TMSP, Sigma-Aldrich, 98 atom % D, CAS: 24,493–21-8) as the chemical shift reference standard) to reach a final volume of 600 µL. In the *two-filter approach*, two filters were used per sample (≈ 350 µL OF each) and the resulting filtrates (≈ 200 µL each) were combined to obtain ≈ 400 µL of filtrate prior to addition of a 200-µL NMR buffer to reach a final volume of 600 µL. For all samples from both approaches, 550 μL was then transferred into 5-mm NMR tubes for analysis.

#### Protein precipitation

After thawing eight frozen ovarian fluid QC samples on ice for ≈ 2.5 h, methanol pre-chilled on ice (800 μL) was added to 400 μL of sample (2:1, v/v). After vortexing the resulting suspension for 10 s, it was incubated at − 20 °C for 10 min. Proteins were pelleted at 14,000 × *g* for 5 min at 4 °C. The supernatant was transferred into new pre-labeled microcentrifuge tubes. Samples were dried using a vacuum centrifuge (Eppendorf, Hauppauge, NY, USA) overnight at room temperature. Dried metabolites were rehydrated with 600 μL of NMR phosphate buffer and vortexed for 10 s until completely redissolved. Samples were centrifuged at 14,000 g at 4 °C for 5 min to pellet any particulates. Subsequently, 550 μL of the samples was transferred into 5-mm NMR tubes for analysis.

#### Lyophilization

Eight frozen 1-mL ovarian fluid QC samples were taken from the − 80 °C freezer and lyophilized (Lyophilizer Heto Cooling Trap) overnight. The following morning, the freeze-dried samples were reconstituted in 600 μL of NMR phosphate buffer, vortexed for ≈ 2.5 min until completely redissolved, and centrifuged at 10,621 × *g*, at 4 °C for 10 min to pellet any particulate matter. A slimy film was observed on the surface of all samples; and therefore, the samples were subjected to an additional centrifugation step. This additional step failed to displace the film. Taking care to avoid touching the slimy film with the pipette tip, 550 μL of the samples was transferred into 5-mm NMR tubes for analysis. Two of the eight samples were used to test the *lyophilization* + *filtration* method (see method below).

#### Dilution

After thawing eight frozen ovarian fluid QC samples on ice for ≈ 2.5 h, samples were centrifuged at 10,621 × *g*, at 4 °C for 10 min to pellet particulates. Four hundred microliters of supernatant per sample was transferred into new pre-labeled microcentrifuge tubes and 200 μL of NMR phosphate buffer was added to a final volume of 600 μL. Subsequently, 550 μL of the samples was transferred into 5-mm NMR tubes for analysis. Two of the eight samples were used to test the *dilution* + *filtration* method (see method below).

The dilution + filtration, as well as the lyophilization + filtration method below, tested the addition of a filtration step during the sample preparations, as follow-up methods to both dilution and lyophilization.

#### Dilution + filtration

From the remaining two samples that underwent dilution, 500 μL per sample was loaded onto centrifugal filters (3-kDa MW cutoff) that had been pre-washed overnight. The filtration protocol (two-filter method) was then followed to obtain 400 μL of filtrates per sample. NMR phosphate buffer (200 μL) was added to the 400 μL samples to a final volume of 600 μL, and vortexed for 60 s. Samples (550 μL) were transferred into 5-mm NMR tubes for analysis.

#### Lyophilization + filtration

From the remaining two samples that underwent lyophilization, 500 μL was loaded onto Nanosep centrifugal filters (3-kDa MW cutoff) (Pall Life Sciences, Port Washington, NY, USA) that had been pre-washed overnight (see *filtration* method for details). The filtration protocol (two-filter method) was then followed to obtain 400 μL of filtrates per sample. NMR phosphate buffer (200 μL) was then added to the 400-μL samples to a final volume of 600 μL, and the samples were vortexed for 60 s. Next, 550 μL of each sample was transferred into 5-mm NMR tubes for analysis.

#### Filtration + concentration

Eight Nanosep centrifugal filters (3-kDa MW cutoff) (Pall Life Sciences, Port Washington, NY, USA) were pre-washed overnight (see *filtration* method for details). Eight aliquots of pooled ovarian fluid control material were thawed on ice for ≈ 2.5 h. Then, 500 μL per sample was loaded onto the centrifugal filters (two-filter method) and centrifuged at 10,621 × *g* at 4 °C to obtain at least 400 μL of filtrate from each sample (≈ 3 h). The filtrates from two filters (400 μL each) were combined into new microcentrifuge tubes to obtain four 800-μL samples. Samples were dried overnight at room temperature using a vacuum centrifuge (Eppendorf, Hauppauge, NY, USA). Dried metabolites were reconstituted in 600 μL of NMR phosphate buffer, vortexed for 60 s until completely redissolved, and centrifuged at 10,621 × *g*, at 4 °C for 10 min to pellet particulates. Samples (550 μL) were transferred into 5-mm NMR tubes (Bruker Biospin, Inc., Billerica, MA, USA) for analysis.

### ^1^H NMR spectroscopy data acquisition

Fish ovarian fluid NMR spectra were acquired at a temperature of 298 K on a Bruker Avance II 700 MHz NMR spectrometer equipped with a 5-mm triple-resonance TXI room-temperature probe and a SampleJet automatic sample changer (Bruker Biospin, Inc., Billerica, MA, USA). One-dimensional (1D) ^1^H NMR spectra were acquired under automation using ICON-NMR (Bruker Biospin) with water suppression using a three-pulse sequence based on a standard one-dimensional (1D) nuclear Overhauser effect spectroscopy (NOESY) pulse sequence with presaturation (pulse program “*noesygppr1d*”). The NMR protocol included 10 min for temperature equilibration, automated shimming with on-axis and off-axis shims, automated probe tuning, and pulse calibration on each individual sample. Spectra were acquired with a spectral width of 20.0 ppm, a relaxation delay of 3.0 s, 200 scans, and eight dummy scans, for a total of 65,536 data points. A 60-ms mixing period was used for solvent suppression and an acquisition time of 2.34 s for a total repetition time (D1 + AQ) of 5.34 s. The receiver gain parameter (RG) was set to 32. The resulting spectra were processed by zero-filling to 65,536 complex points and by multiplying the free induction decay (FID) by an exponential-line-broadening function of 0.3 Hz prior to Fourier transformation. The resulting spectra were calibrated to the TMSP peak set at 0.0 ppm, automatically phased, and baseline-corrected by applying a fifth-order polynomial with additional manual phase correction when necessary using the TopSpin 3.5 software (Bruker Biospin, Inc.).

### ^1^H NMR data processing and multivariate statistical analysis

Processed ^1^H NMR spectra were assessed by visual inspection and were analyzed using multivariate analysis. Prior to statistical analysis, spectra were binned from 0.20 to 10.0 ppm using a 0.005 bin width in NMRProcFlow v1.3.10 (Jacob et al. 2017). The *S*/*N* ratio for each bin was calculated using NMRProcFlow. Bins with *S*/*N* ratio < 3 were removed (denoising), and the water region (4.70 to 4.90 ppm) was excluded, which resulted in 1147 spectral bins for subsequent multivariate statistical analysis. Binned spectra were normalized using the constant total spectral area and the bins were Pareto scaled in which the bin intensity is divided by the square root of the standard deviation of the bins. Data were analyzed using principal component analysis (PCA) and visualized using MetaboAnalyst 5.0 (Chong et al. 2019). Spectral data quality was assessed using the percentage relative standard deviation (% RSD) of individual bins across replicate samples and the median % RSD was reported (Parsons et al. 2009).

## Results and discussion

**Fig. 3:**
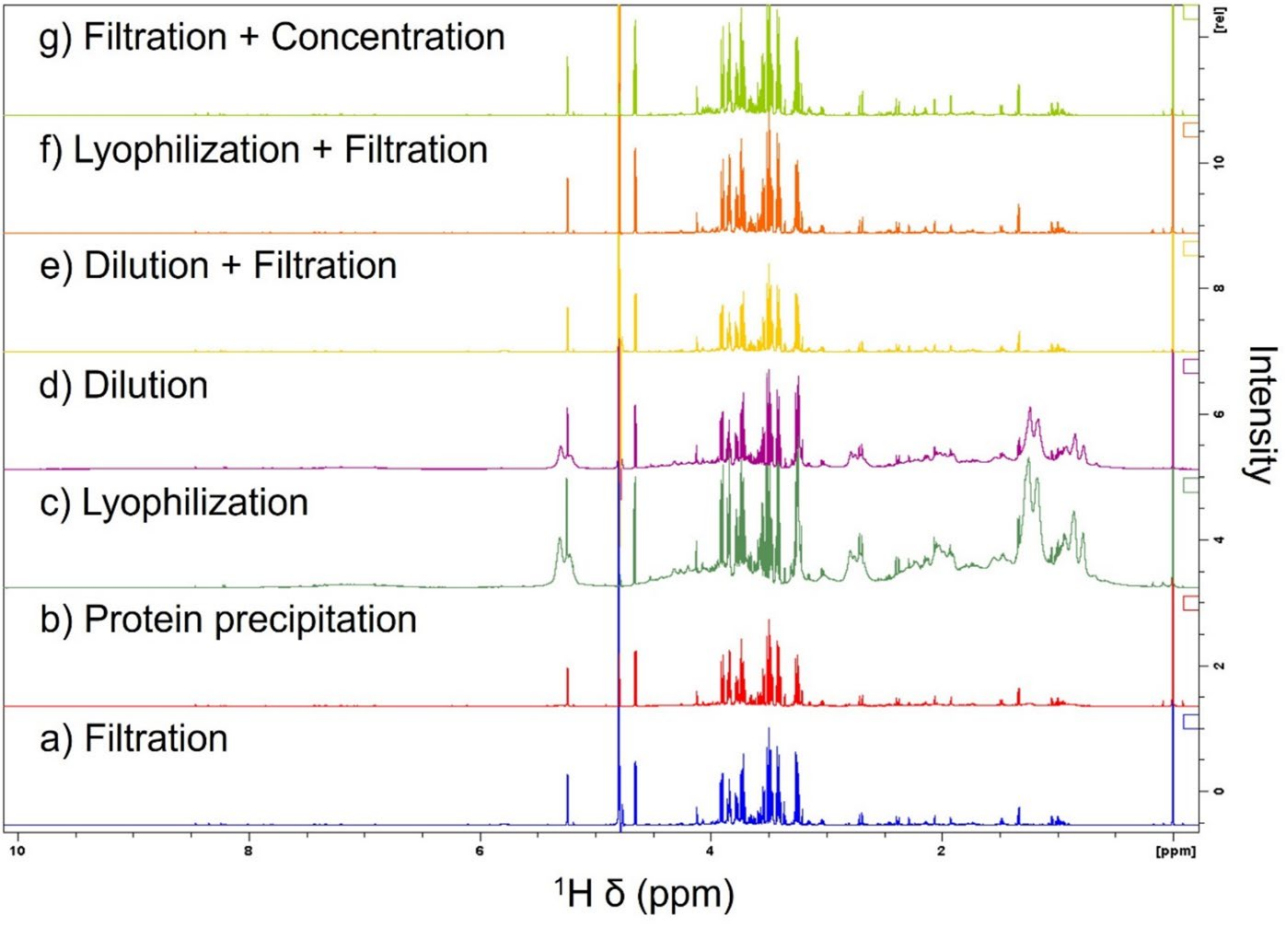
^1^H NMR full spectra comparison of the seven methods tested for Atlantic salmon ovarian fluid processing for metabolomic analysis

Atlantic salmon ovarian fluid (OF) may provide biochemical information that represents the reproductive fitness of an individual fish. Metabolomic characterization of this matrix was conducted using ^1^H NMR spectroscopy. The ^1^H NMR spectra of Atlantic salmon OF for each sample preparation method tested in this study are shown in Fig. 3.

**Fig. 4:**
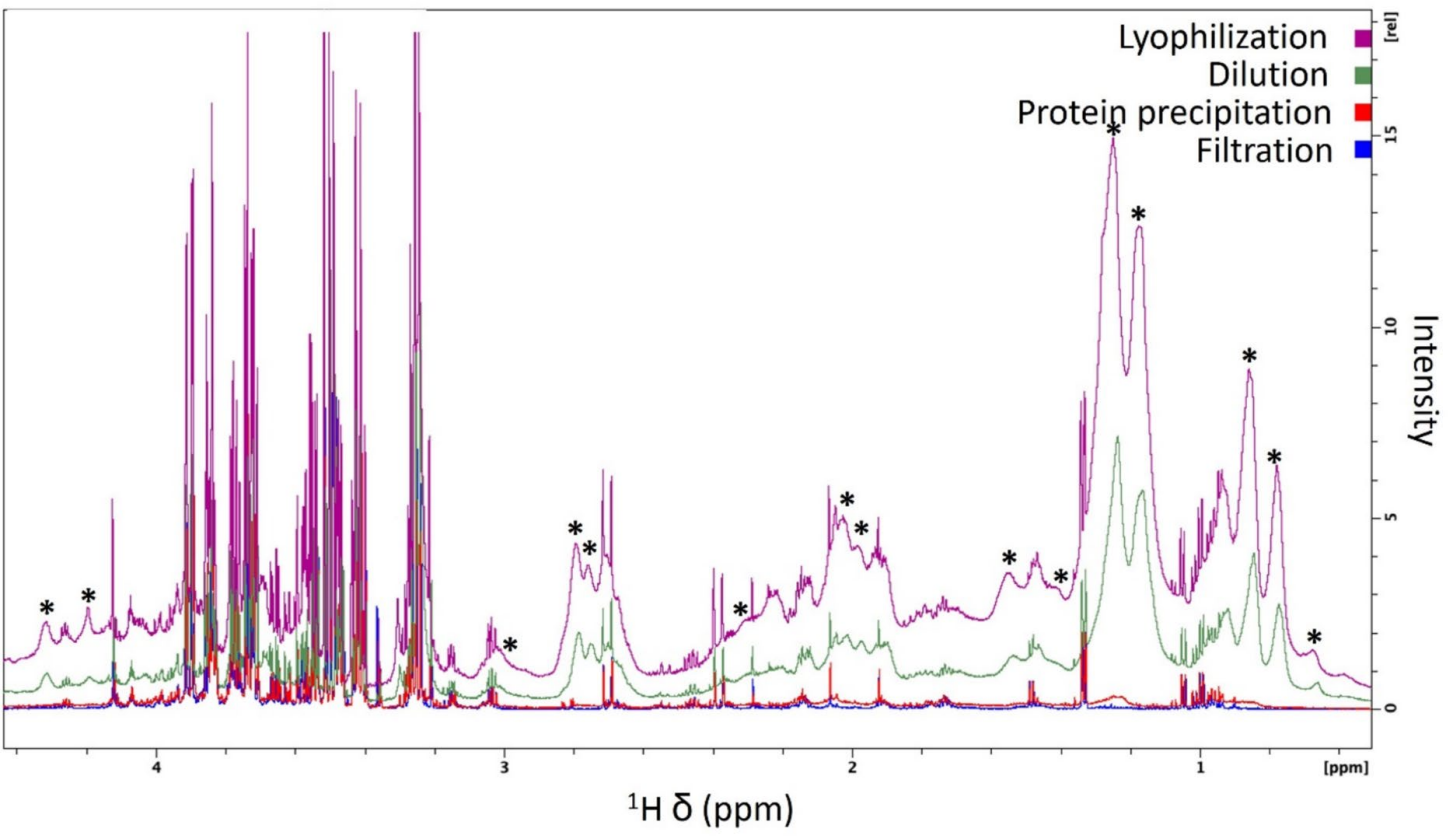
^1^H NMR spectral region (0.6–4.4 ppm) displaying a comparison of four methods for Atlantic salmon ovarian fluid processing showing broad macromolecule peaks in the *lyophilization* (purple), *dilution* (green) and *protein precipitation* (red) methods compared with the *filtration* (blue) method. Asterisks highlight broad peaks causing significant baseline distortion and masking peaks from low-molecular-weight metabolites

We observed pronounced spectral baseline distortion due to the presence of macromolecules (i.e., proteins, lipids) in the samples when using the *dilution* and *lyophilization* methods. These methods do not involve the physical separation (“deproteinization”) of such molecular components from the sample prior to NMR analysis (Fig. 4).

**Fig. 5:**
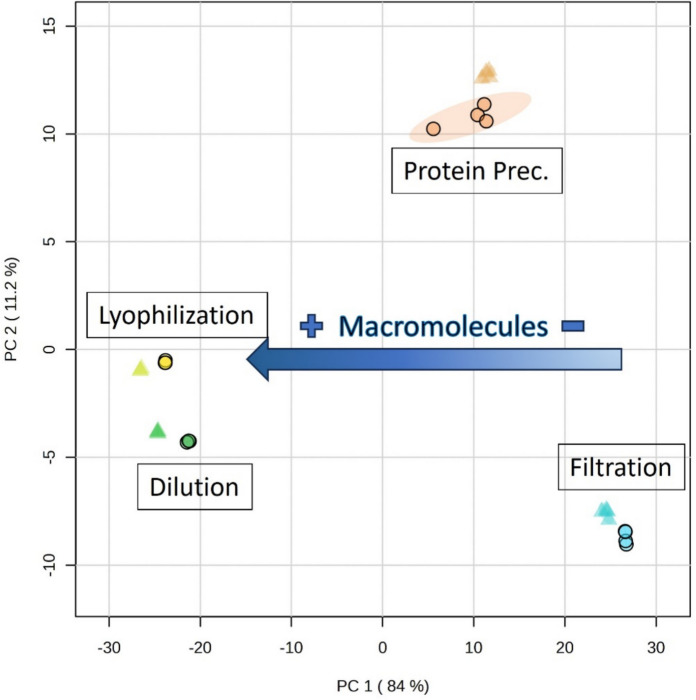
Principal component analysis of ^1^H NMR spectra from four ovarian fluid processing methods. Ellipses represent 95% confidence regions. Ellipses are calculated for all groups but, except for the *protein precipitation* method, they are too small to be visualized. Different blood contamination levels are indicated using different symbols: low blood contamination (circles), high blood contamination (triangles)

In addition, residual macromolecule peaks were observed when using the methanol protein precipitation method at a ratio of 2:1 (v/v) methanol:OF (Figs. 3 and 4). High-molecular-weight molecules negatively affect NMR spectral quality due to the broad peaks originating from them that can completely obscure the peaks arising from the low-molecular-weight metabolites and subsequently impair metabolite identification and relative quantification, thus limiting the amount of obtainable information (McHugh et al. 2018; Yanibada et al. 2018; Daykin et al. 2002). The presence of high levels of macromolecules in the samples obtained using *dilution* and *lyophilization* is reflected in a PCA score plot defining the ^1^H NMR spectral differences from the filtration method (Fig. 5).

Taken together, these observations indicate that the *dilution* and *lyophilization* methods are not ideal for discovery-based metabolomics analyses, where comprehensive and unbiased detection of metabolites is essential. Because of the presence of macromolecules, we decided to test the addition of a filtration step (dilution + filtration (*N* = 2), and lyophilization + filtration (*N* = 2)), to physically remove them from the sample as shown in the ^1^H spectra comparison in Supplementary Figures S1 and S2. The filtration methods are explored further below. In addition, alternative approaches based on macromolecule-signal suppression using NMR pulse sequences such as the Carr-Purcell-Meiboom-Gill (CPMG) T_2_-filtering sequences could be considered. These approaches were not evaluated in this study. Although ^1^H CPMG experiments can provide aid in qualitative identification of metabolites, by attenuating signals from proteins and lipids, they are to be used with caution when accurate metabolite quantitation is needed, since certain metabolites may also experience signal loss, thus leading to inaccurate quantification. Also, the CPMG is generally not considered a quantitative sequence since it can induce non-uniform relaxation of metabolite signals across the spectrum (Mulard et al. 2025).

**Fig. 6:**
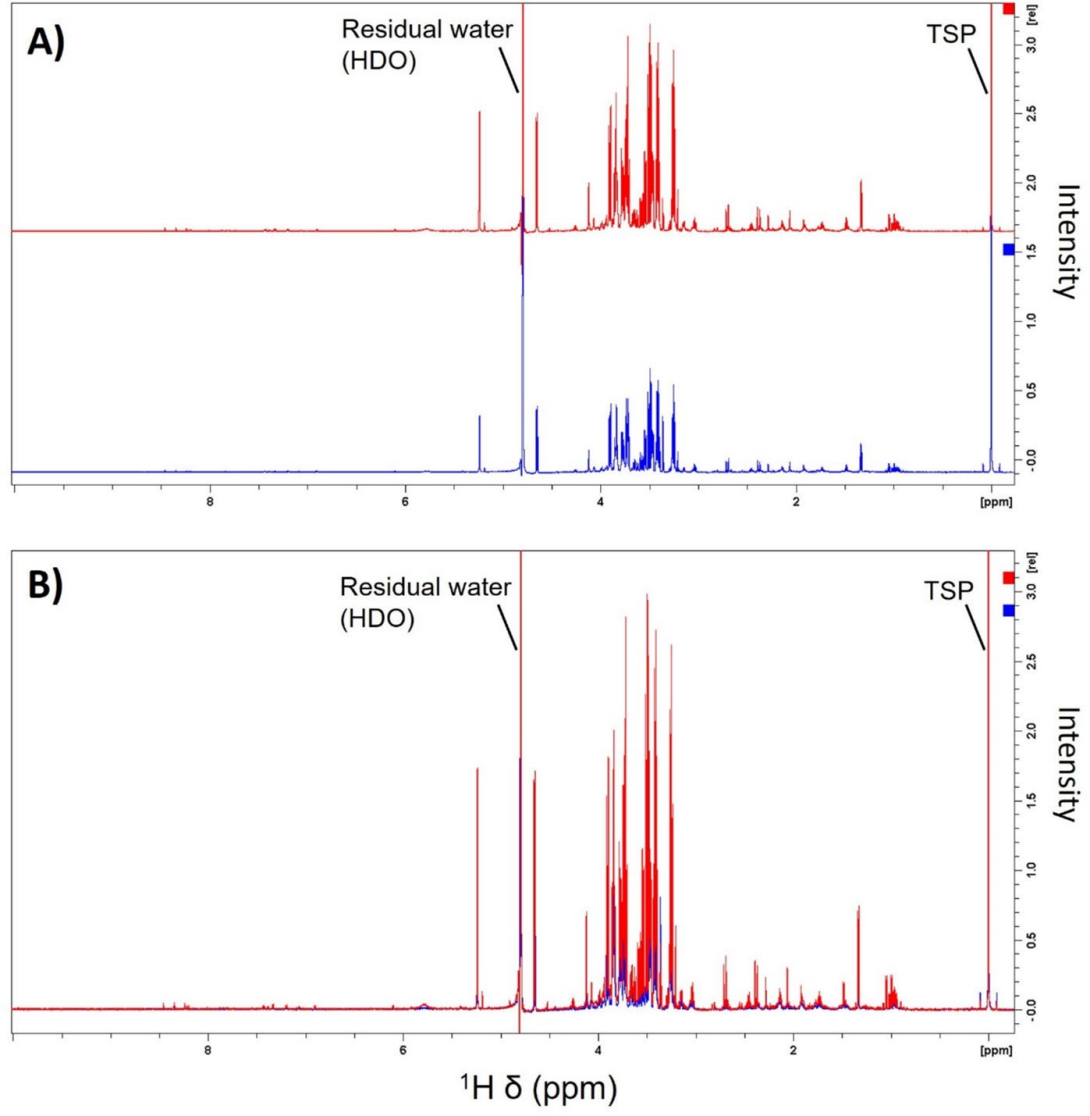
Comparison of ^1^H NMR spectra of Atlantic salmon ovarian fluid obtained from a one-filter (blue) vs. a two-filter (red) approach using the *filtration* method. **A** Stacked and **B** overlaid ^1^H NMR spectra

To further assess the *filtration* method and to evaluate different volumes of filtrate for NMR analysis, we compared the use of a single centrifugal filter to a two-filter-per-sample approach. The use of two filters per sample provided a larger volume of filtrates, thus leading to an improvement in NMR spectral signal-to-noise ratios compared to a single filter. The two-filter method was particularly useful for the detection of metabolites present at low concentrations in the OF (Fig. 6).

This enhancement in spectral quality supports the value of optimizing filtration steps in the sample preparation protocol. The potential for certain metabolites to be present in free form as well as bound to macromolecules (e.g., bound to albumin in blood samples) should be considered in the *filtration* method since the removal of macromolecules by filtration could also cause the loss of metabolites bound to these macromolecules, thereby reducing their concentration in the final NMR sample. Therefore, alternative sample preparation methods may be more suitable for use in targeted metabolomics workflows that are intended for absolute quantitation of specific metabolites of interest. While this study prioritizes the evaluation of different OF sample preparation methods based on overall spectral quality, repeatability, and ease of execution over metabolite quantitation, the inherently quantitative nature of NMR warrants further considerations. In untargeted metabolomics approaches where all detectable metabolites are evaluated, several factors make absolute quantification challenging, including extensive signal overlap within complex biological matrices which leads to metabolite signal masking, the use of short relaxation delays to optimize the signal-to-noise ratio might not allow nuclear relaxation for all nuclei, and the potential for signal loss due to protein binding to small molecules. Furthermore, the lack of purified internal standards for every detectable feature, coupled with the discovery-based nature of an untargeted approach, renders the absolute quantification of all detected metabolites technically prohibitive.

**Fig. 7:**
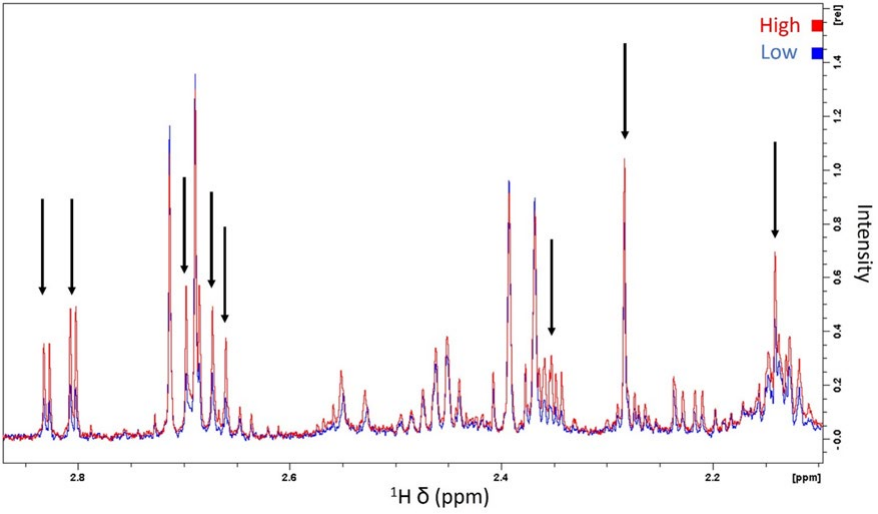
Influence of blood contamination levels on ^1^H NMR metabolite profiles of Atlantic salmon ovarian fluid (*filtration* method). Differences in intensity can be observed for several peaks (black arrows), which indicate differences in concentration for various metabolites between high (red spectrum) and low (blue spectrum) blood contamination of ovarian fluid samples

A confounding factor to consider is blood contamination during sample collection. Blood contamination during OF collection in fish is a common issue that can impact downstream analyses and experiments. This can occur particularly when sample collection involves stripping, a procedure that can inadvertently damage small blood vessels, thus resulting in blood release into the ovarian fluid (Dietrich et al. 2008; Lahnsteiner et al. 1995; İnanan and Öğretmen 2015). Our results demonstrate that blood contamination of OF samples alters their metabolite profiles as summarized in the PCA score plot in Fig. 5, where the samples with higher blood contamination levels show distinct metabolomic profiles in terms of relative metabolite levels from those with lower blood contamination levels, regardless of the method used. The influence of blood contamination levels on metabolite peak intensities is shown for the spectral subregion between 2.10 and 2.85 ppm (Fig. 7), with a general increase in peak intensities detected in the samples with a high blood contamination level compared with those with low blood contamination.

These results reinforce the importance of minimizing blood contamination during OF collection to ensure biologically meaningful metabolomic comparisons. Our observations may not preclude the use of blood-contaminated OF samples, but further investigations are needed to evaluate the effect of blood contamination levels on the concentration of metabolites/biomarkers of interest identified using other methods.

**Fig. 8:**
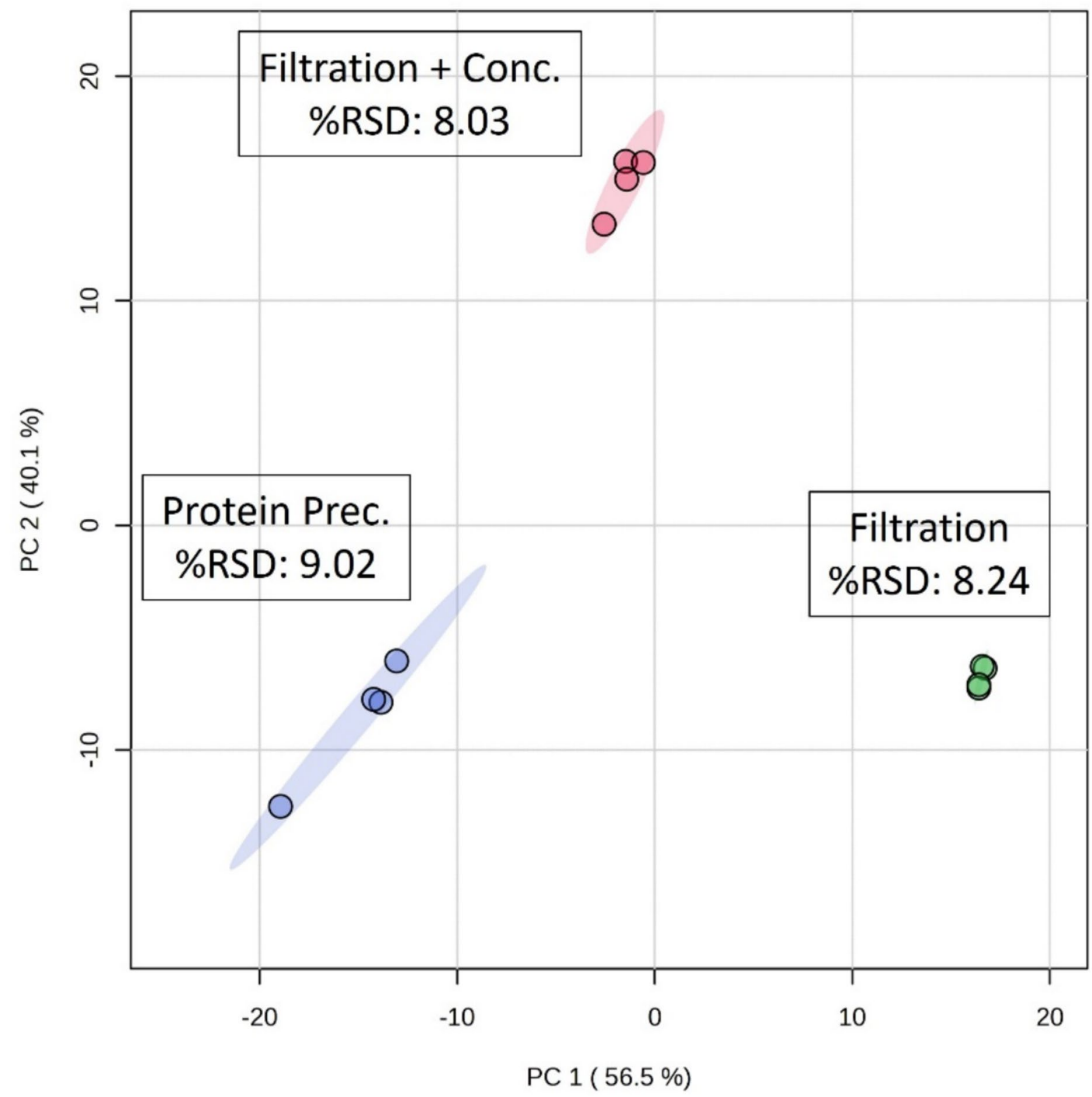
PCA scores plot of *protein precipitation*, *filtration*, and *filtration* + *concentration* preparation methods for salmon ovarian fluid. Ellipses represent 95% confidence regions for each group. Spectral median % RSD values are indicated for each method

Subsequent analyses were performed exclusively on non-contaminated OF samples. A comparative PCA score plot calculated using ^1^H NMR spectra of OF for *protein precipitation*, *filtration*, and *filtration* + *concentration* is displayed (Fig. 8).

Explained variance (EV) were 56.5% for PC1 and 40.1% for PC2 with a total EV for PC1-PC2 of 96.6%. Overall, the *filtration* + *concentration* method showed a lower value of spectrum-wide variability with a median % RSD of 8.03% comparable to the *filtration* only method that had a median % RSD of 8.24%, whereas the *protein precipitation* method had a higher median % RSD of 9.02%. Although the difference in median % RSD is relatively small, the presence of residual macromolecules in the samples after protein precipitation constitutes a drawback for spectral quality. While reporting the median % RSD conveys the repeatability across the whole spectrum, it is essential to understand the measurement quality of individual peaks/bins. The % RSD for individual bins was calculated to visualize the variation within each extraction method. Out of 1147 spectral bins, the method with the highest percentage of bins with RSD < 10% was the *filtration* + *concentration* method suggesting that this method had the least amount of variation (Table 1). Spectral signal-to-noise (*S*/*N*) ratios were also evaluated. The *filtration* + *concentration* provided the highest *S*/*N* value (33.2) despite a longer processing time with this method due to the addition of an overnight concentration step.

**Table 1:** Comparison of ovarian fluid sample preparation methods tested in this study

Method	^a^Residual macromolecules	Median % RSD	% Bins with RSD < 10%	^b^Signal-to-noise (*S*/*N*)	Processing time (h)
Filtration	No	8.24	56.8	20.3	≈ 6
Protein precipitation	Yes	9.02	56.0	21.3	≈ 14
Lyophilization	Yes	N/A	N/A	N/A	> 24
Dilution	Yes	N/A	N/A	N/A	≈ 3
Filtration + concentration	No	8.03	59.0	33.2	≈ 14
^c^Dilution + filtration	No	N/A	N/A	19.9	≈ 9
^c^Lyophilization + filtration	No	N/A	N/A	26.3	> 24

^a^Residual macromolecules from ^1^H NMR spectra

^b^*S*/*N* was calculated using NMRProcFlow

^c^For these methods, *N* = 2 and no statistical inferences were made

**Fig. 9:**
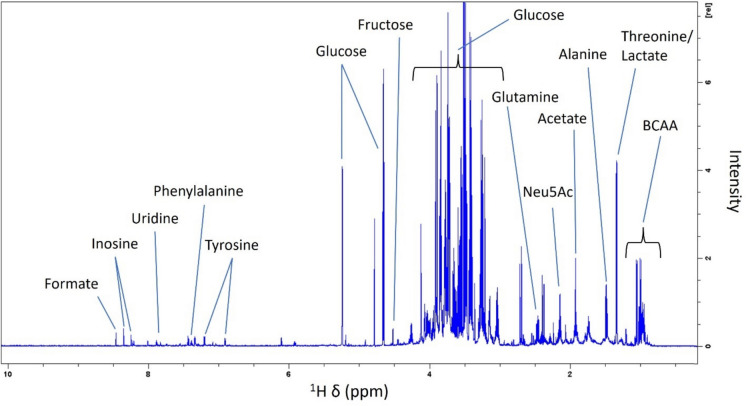
Partially annotated ^1^H NMR spectrum of Atlantic salmon ovarian fluid obtained using the *filtration* + *concentration* preparation method. *Neu5Ac* N-acetylneuraminic acid, *BCAA* branched-chain amino acids

A list of 45 metabolites detected in OF samples is provided in Supplementary Table S2. In addition, a partially annotated spectrum of Atlantic salmon ovarian fluid obtained using the *filtration* + *concentration* method is shown in Fig. 9.

## Conclusions

In summary, fish ovarian fluid (OF) constitutes a viable matrix for the measurement of metabolites using NMR-based metabolomics. Supplies needed for ovarian fluid collection are minimal, easily obtainable from commercial sources, and affordable. Sample collection is simple, rapid, and minimally invasive compared to blood and tissue collection, which suggests its reproducibility across different operators and facilities. Our results highlight the importance of minimizing accidental blood contamination that can occur during sample collection for reliable metabolite measurements and interpretation of outcomes. Future work on OF may incorporate quantitative hemoglobin measurements; for example, a spectrophotometric assay to measure hemoglobin absorbance at a frequency of ≈400 nm could be used to correlate total hemoglobin concentration in individual OF samples to changes in specific metabolite concentrations. OF’s chemical composition is quite complex, and similar to other matrices such as blood, it is characterized by the presence of macromolecules (proteins, lipids) which can interfere with metabolite measurements. To analyze low-molecular-weight metabolites in metabolomic analysis, it is recommended to remove macromolecules during sample processing since they generate broad signals that can obscure the narrow peaks arising from the small metabolites and interfere with metabolite identification and quantification. Although any sample preparation method for untargeted metabolomic analyses will inevitably introduce bias, our evaluation criteria emphasized NMR spectral quality and method reproducibility and practicality rather than the effect of different preparation methods on individual metabolites. It remains critical that biological interpretations are framed within the context of the specific metabolome analyzed by the chosen methodology. Overall, our results show that *filtration* + *concentration* was the best method among those tested in this study based on the low % RSD, higher *S*/*N*, and ease of execution. This method allows for the evaluation of OF metabolite profiles across different broodstock experimental groups and can aid in the identification of fish reproductive biomarkers, as well as provide a foundation for standardized NMR metabolomic studies in salmon broodstock reproductive research.

## Supplementary information

Below is the link to the electronic supplementary material.

ESM 1 (DOCX 319 KB)

## Data Availability

All data supporting the findings of this study are available within the paper.

## Declarations

### Disclaimer

Certain commercial equipment, instruments, trade names, products, or materials are identified in this paper in order to specify the experimental procedure adequately. Such identification is not intended to imply or convey and should not be interpreted as conveying official approval, recommendation or endorsement by the National Institute of Standards and Technology (NIST), nor is it intended to imply that the materials or equipment identified are necessarily the best available for the purpose.
