# Supplementary material for: Evaluation of sample preparation methods for NMR-based metabolomics of Atlantic salmon (Salmo salar) ovarian fluid
Source: Fish Physiol Biochem. 2026 Apr 15;52(2):63. doi: 10.1007/s10695-026-01678-0 (PMC13083381; doi:10.1007/s10695-026-01678-0)
Supplement: Supplementary file 1 — (DOCX 319 KB) [file 10695_2026_1678_MOESM1_ESM.docx]

**Supplementary Information:**

**Evaluation of sample preparation methods for NMR-based metabolomics of Atlantic Salmon (*Salmo salar*) ovarian fluid**

Fabio Casu^a*^, Amanda L. Bayless^a^, Brian C. Peterson^b^, Heather J. Hamlin^c,d^, Ashley S. P. Boggs^a^, and Tracey B. Schock^a^

^a^National Institute of Standards and Technology (NIST), Chemical Sciences Division, Hollings Marine Laboratory, Charleston, SC 29412, USA

^b^USDA Agriculture Research Service, National Cold Water Marine Aquaculture Center, 25 Salmon Farm Road, Franklin, ME 04634, USA

^c^Aquaculture Research Institute, 17 Godfrey Dr., University of Maine, Orono, ME 04473, USA

^d^School of Marine Sciences, 360 Aubert Hall, University of Maine, Orono, ME 04469, USA

^*^Corresponding author. National Institute of Standards and Technology (NIST), Chemical Sciences Division, Hollings Marine Laboratory, Charleston, SC 29412, USA. *E-mail*: fabio.n.casu@nist.gov


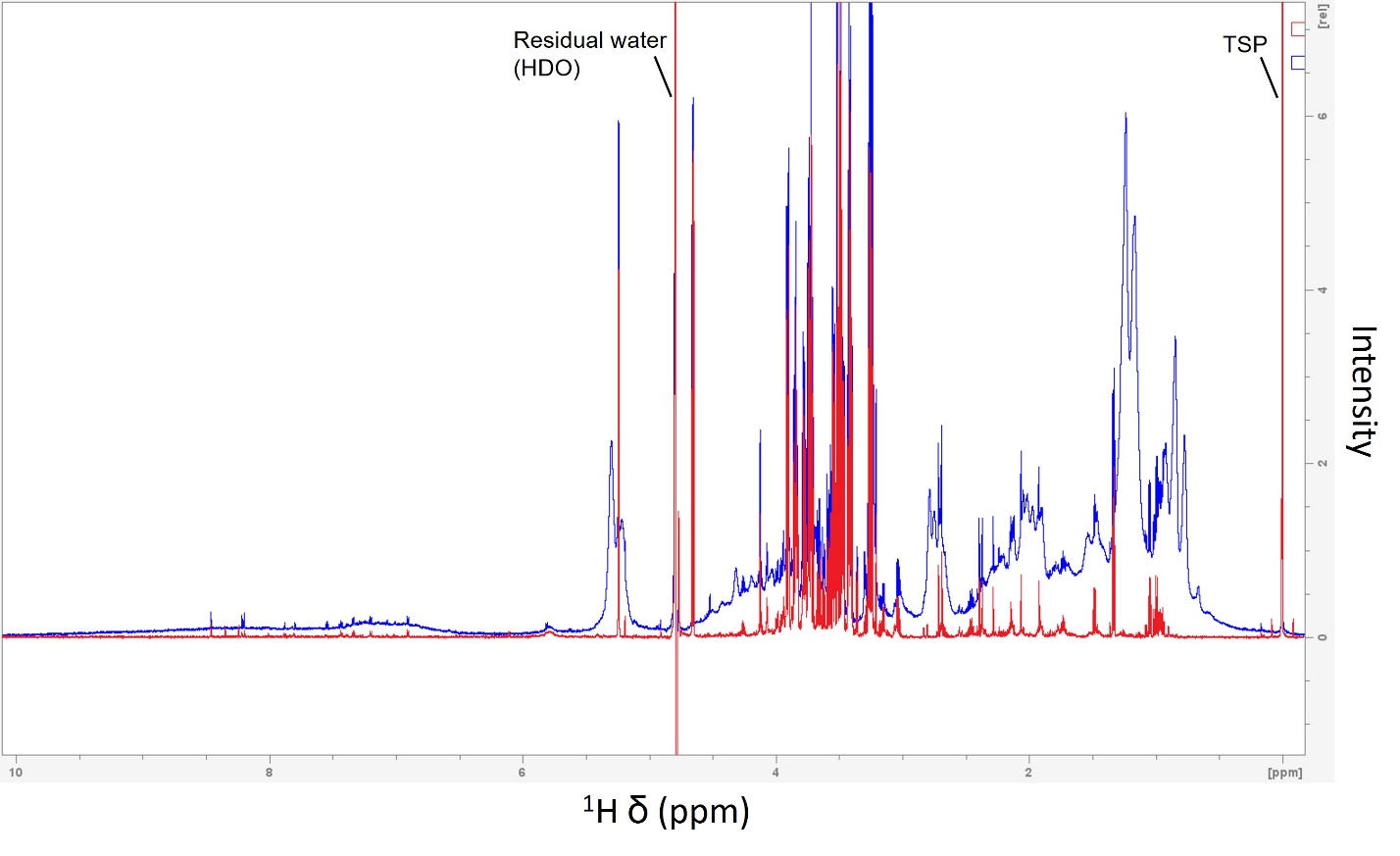


**Fig. S1** ^1^H NMR spectra displaying a comparison of the *Lyophilization* and *Lyophilization + Filtration* methods for Atlantic salmon ovarian fluid processing. Broad macromolecule peaks can be observed in the *Lyophilization* (blue), compared with the *Lyophilization + Filtration* (red) method


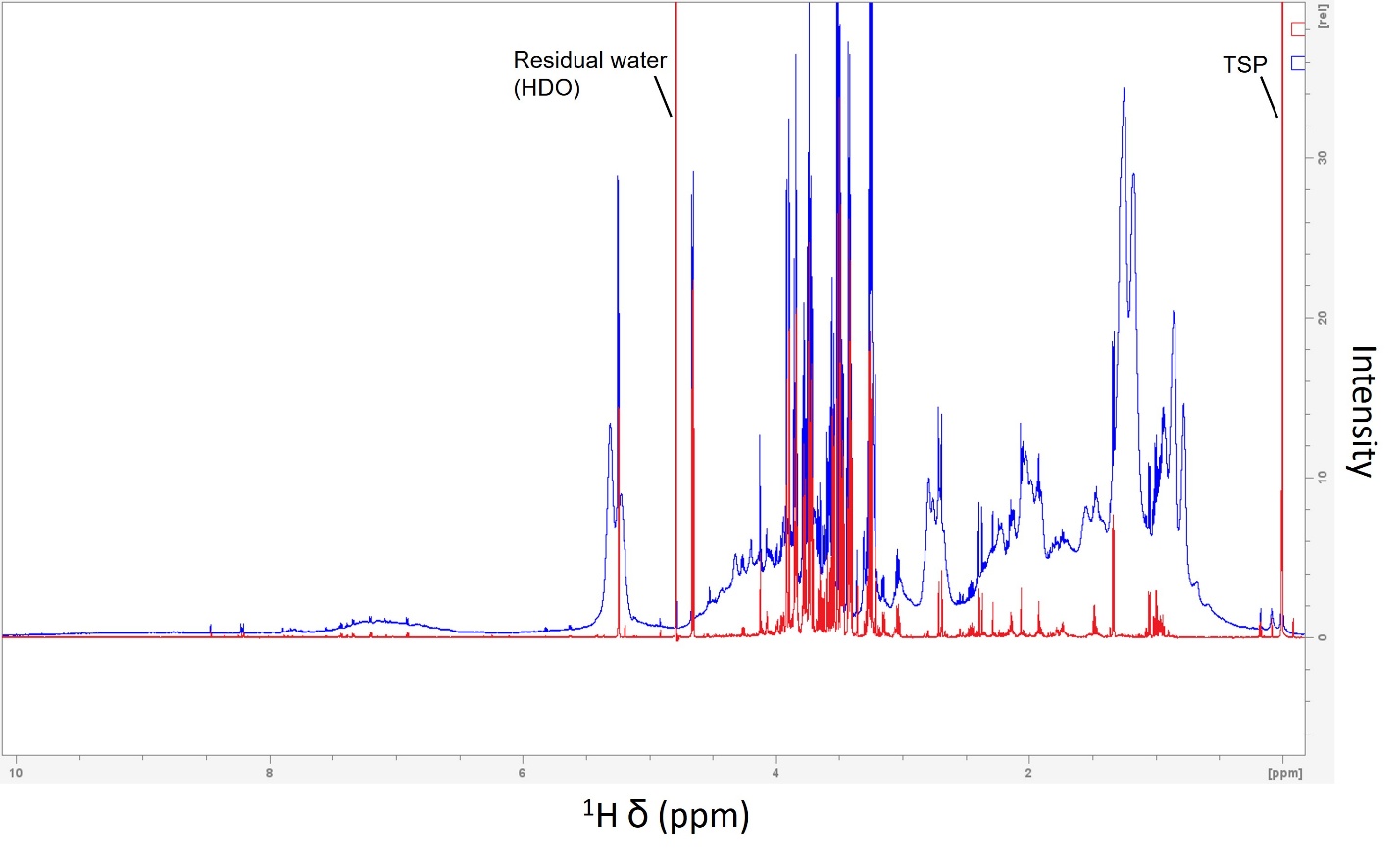


**Fig. S2** ^1^H NMR spectra displaying a comparison of the *Dilution* and *Dilution + Filtration* methods for Atlantic salmon ovarian fluid processing. Broad macromolecule peaks can be observed in the *Dilution* (blue) method, compared with the *Dilution + Filtration* (red) method

**Table S1** Overview of OF aliquots, sample and NMR buffer volumes for each method tested in this study

| Ovarian Fluid (OF) Preparation Method | Frozen Aliquots Used | Frozen Aliquot Volume  (µL) | StartingOF Volume  (µL) | Resulting  OF Volume  (µL) | NMR Buffer  (µL) | Final NMR Sample Volume  (µL) |
| --- | --- | --- | --- | --- | --- | --- |
| Filtration (One Filter) | 4 | 400 | 350 | 200 | 400 | 550 |
| Filtration (Two Filters) | 4 | 400 (x2) | 350 (x2) | 400 | 200 | 550 |
| Protein Precipitation | 8 | 400 | 400 | ^a^dried | 600 | 550 |
| Lyophilization | 6 | 1000 | 1000 | ^a^dried | 600 | 550 |
| Dilution | 6 | 400 | 400 | 400 | 200 | 550 |
| Dilution  Lyophilization + Filtration | 2 | 400 | 400 | 400 | 200 |  |
| + Filtration |  |  | 500 | 400 | 200 | 550 |
| Lyophilization | 2 | 1000 | 1000 | ^a^dried | 600 |  |
| + Filtration |  |  | 500 | 400 | 200 | 550 |
| Filtration | 8 | 400 | ^b^500 | 400 (x2) | - | - |
| + Concentration |  | - | - | ^a^dried | 600 | 550 |

^a^For these methods, the sample was dried and then reconstituted in NMR buffer.

^b^An extra 400 µL aliquot was used to obtain 500 µL starting volume.

**Table S2** List of 45 common metabolites detected in salmon ovarian fluid samples from each method tested in this study. Corresponding metabolite class and biological relevance are shown for individual metabolites

| Metabolite | Metabolite Class | Biological Relevance |
| --- | --- | --- |
| Acetate | Organic acids | Lipid metabolism |
| Acetoacetate | Ketone bodies | Lipid metabolism |
| Acetone | Ketone bodies | Lipid metabolism |
| Alanine | Amino acids | Energy metabolism |
| Arginine | Amino acids | Nitrogen metabolism |
| Ascorbate (Vit. C) | Vitamins | Enzyme cofactor |
| Aspartate | Amino acids | Protein synthesis |
| Betaine | Osmolytes | Osmoregulation |
| Carnitine | Amino acid derivatives | Lipid transport |
| Choline | Choline and derivatives | Lipid metabolism |
| Creatine | Organic acids | Energy metabolism |
| Ethanolamine | Phospholipids and derivatives | Lipid metabolism |
| Formate | Organic acids | Energy metabolism |
| Fructose | Carbohydrates | Energy metabolism |
| Glucose | Carbohydrates | Energy metabolism |
| Glutamate | Amino acids | Neurotransmission |
| Glutamine | Amino acids | Nitrogen transport |
| Glutathione | Peptides | Antioxidant |
| Glycerol | Sugar alcohols | Energy metabolism |
| Glycine | Amino acids | Protein metabolism |
| Hypoxanthine | Purine metabolites | Nucleotide metabolism |
| Inosine | Purine metabolites | Nucleotide metabolism |
| Isoleucine | Branched-chain amino acids | Energy metabolism |
| Lactate | Organic acids | Anaerobic metabolism |
| Leucine | Branched-chain amino acids | Protein metabolism |
| Lysine | Amino acids | Protein metabolism |
| Malate | Organic acids | TCA cycle |
| Mannose | Carbohydrates | Energy metabolism |
| Methionine | Amino acids | Protein metabolism |
| *myo*-Inositol | Sugar alcohols | Lipid metabolism |
| N-acetylneuraminic acid | Sialic acids | Glycoprotein metabolism |
| Ornithine | Amino acid derivatives | Urea cycle |
| Phenylalanine | Amino acids | Protein metabolism |
| Putrescine | Polyamines | Polyamine metabolism |
| Pyridoxine (Vit. B6) | Vitamins | Enzyme cofactor |
| Pyroglutamate | Pyrrolidines | Glutathione metabolism |
| *scyllo*-Inositol | Sugar alcohols | Osmoregulation |
| Serine | Amino acids | One-carbon metabolism |
| Succinate | Organic acids | TCA cycle |
| Taurine | Amino acid derivatives | Osmoregulation |
| Threonine | Amino acids | Protein metabolism |
| Tryptophan | Amino acids | Protein metabolism |
| Tyrosine | Amino acids | Protein metabolism |
| Uridine | Nucleosides | RNA metabolism |
| Valine | Branched-chain amino acids | Energy metabolism |
|  |  |  |
